# MicroRNAs as Biomarkers of Cervical Cancers

**DOI:** 10.3390/ijms27125330

**Published:** 2026-06-12

**Authors:** Wojciech Jelski, Sylwia Okrasinska, Jan Mroczko, Weronika Rutkowska, Klaudia Zieziula, Barbara Mroczko

**Affiliations:** 1 Department of Biochemical Diagnostics, Medical University, 15-269 Bialystok, Poland; 2 Department of Biochemical Diagnostics, University Hospital, 15-269 Bialystok, Poland; 3 Department of Neurodegeneration Diagnostics, Medical University, 15-269 Bialystok, Poland

**Keywords:** microRNA, cervical cancer, biomarker

## Abstract

Invasive cervical cancer is a very common cause of cancer death in women worldwide, primarily due to late detection of this cancer. The clinical manifestations of cervical cancer vary significantly and are difficult to predict. Finding new effective biomarkers for the early detection of cervical cancer is essential to reducing mortality. Small microRNA molecules have also recently emerged as potential biomarker candidates in the diagnosis of cervical cancer. Despite analytical limitations in microRNA assays and the lack of automated and standardized tests, validated and prospective systematic evaluation of this new parameter in cervical cancer deserves further development. This review describes the importance and potential usefulness of microRNAs in detecting cervical cancer at an early stage, monitoring the course of the disease, and assessing the effectiveness of treatment. The diagnostic importance of microRNAs is well documented in many publications, suggesting that, as microRNA research progresses, they may become a useful diagnostic tool for cervical cancer.

## 1. Introduction

Cervical cancer (CC) is the fourth most common malignancy (after breast, colon, and lung cancer) and the fourth leading cause of cancer deaths in women worldwide. In 2022, an estimated 660,000 new cases of cervical cancer and approximately 350,000 deaths were reported worldwide [1]. Among women of reproductive age, CC ranked among the top third most common cancers and causes of cancer deaths in over 150 countries. If 2022 rates do not decrease, the global burden of CC is estimated to increase to over 760,000 new cases (about 18%) by 2030. Significant geographic variations in cervical cancer incidence are observed across regions of the world. Highly developed countries have incidence rates half as low and mortality rates five times lower than those in low-developed countries. Thus, cervical cancer remains a significant challenge for 21st-century medicine worldwide, especially since it is largely preventable through effective therapeutic approaches [2]. Most CC cases (about 99%) are associated with the oncogenic human papillomavirus (HPV). The most common HPV type in cervical cancer is HPV-16, followed by HPV-18, HPV-45, and HPV-31 [3]. The process of carcinogenesis from normal epithelium to invasive cervical cancer can take more than a decade. In early stages I and II, the total survival rate for women with CC is 72–91%; however, in stage IVa, this rate drops sharply to about 16%. This high mortality rate from cervical cancer is due to its asymptomatic and nonspecific nature in the early stages, which significantly complicates early detection [4]. Detecting early stages of the disease with a good prognosis is not easy. Currently, there is no standardized, reliable screening test due to the complex molecular biology of oncogenesis in cervical cancer. Understanding the biomolecular pathomechanisms of CC development could lead to the development of more reliable and sensitive screening tests and the discovery of more effective treatment methods. Therefore, early detection plays an important role in a positive prognosis for cervical cancer and a reduction in the burden of the disease. Therefore, finding more effective diagnostic and predictive tools in the form of new biomarkers is a significant research challenge in this field. Biomarkers are “biological molecules found in various body fluids or tissues that are a sign of a normal or abnormal process, or of a health condition or disease,” [5] such as cancer. They have numerous potential applications in oncology, such as screening, risk assessment, differentiation, prognosis assessment, and monitoring of cancer progression and response to treatment [5]. Recently, microRNAs were discovered to be involved in the pathomechanism of various types of cancers studied, primarily through abnormal gene expression. MicroRNA profiling has enabled the identification of signatures associated with the diagnosis, prognosis, and response to treatment in human cancers, including cervical cancer [6].

**Figure 1:**
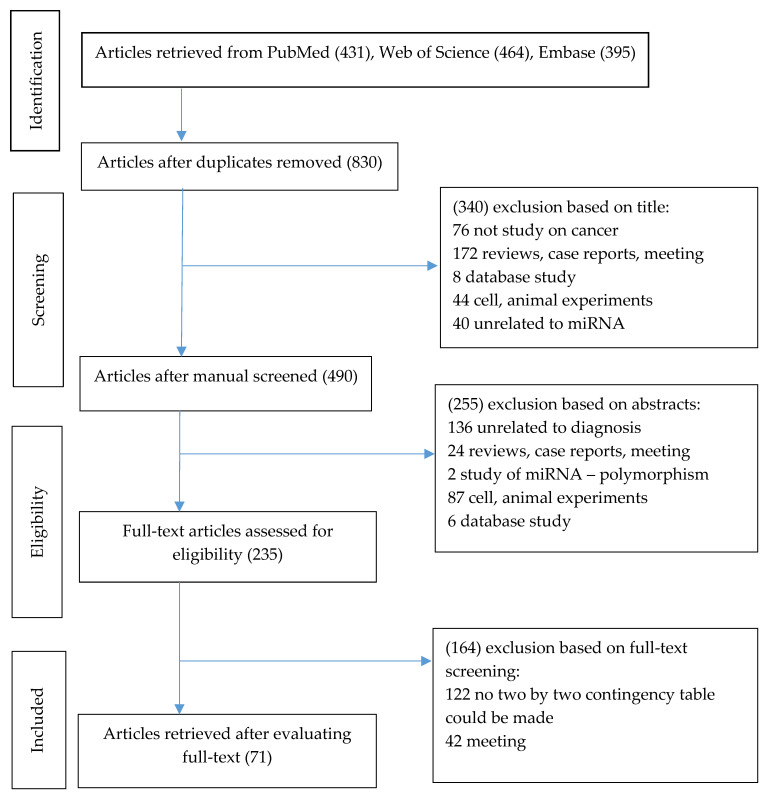
Flow diagram of the article selection for the meta-analysis.

In this systematic review, we analyze the diagnostic usefulness of microRNAs in cervical cancer. The authors conducted a detailed analysis of articles from databases such as Web of Science, Embase, and PubMed. The articles were collected between 2012 and 2025, with particular emphasis on the last 10 years. The joint analysis of the three databases showed positive results, with an overall recovery rate of over 85%. Ultimately, published reviews were included, yielding a total of 1290 relevant references in our databases (Figure 1).

The search terms used were “cervical cancer,” “microRNAs,” “biomarkers,” and “diagnostic utility.” We have adopted inclusion criteria for useful publications, which were analyzed as part of our full-text review: publications addressing the association between the concentrations of the microRNAs studied and the diagnosis of cervical cancer. The research was assessed as useful for specific criteria: reported diagnostic ability of a specific microRNA to detect cervical cancer; and cancer diagnosis was confirmed by histological analysis. Exclusion criteria included: animal studies; publications not in English; letters and meeting records; insufficient or unavailable data; duplicate studies; and studies involving women undergoing radiotherapy.

## 2. Biological Role of microRNAs in Cervical Cancer

MicroRNAs are short, non-coding RNAs that alter gene expression by cleaving the microRNA or slowing down its translation [7]. Physiological microRNAs are single-stranded RNAs approximately 18–25 nucleotides long, sequentially processed from the primary transcript (pri-microRNA) and the resulting stem–loop structure (pre-microRNA) by the Drosha and Dicer proteins, respectively [8]. MicroRNAs exist as several subtypes, such as long non-coding RNAs (lncRNAs), microRNAs (microRNAs), and piwi-interacting RNAs (piRNAs). The modulating roles of these microRNAs are different, and the differences result from different types. One of their functions involves epigenetic changes, as well as protein degradation, which underpins their physiological functions. Therefore, deregulation of these non-coding RNAs is associated with cancer development, its initiation, and its progression. Many microRNAs are tissue- or differentiation-specific, and their temporal expression alters gene expression. The mechanism involves pairing complementary nucleotide sequences of the target sequence [9]. Human microRNAs have been found to be frequently present in sensitive places and chromosomal parts affected by cancer. Thus, chromosomal changes are the main mechanism underlying altered microRNA expression in cancer, as has already been demonstrated in different cancers [10]. Some microRNAs can be transported through exosomes and microvesicle bodies into the blood, among others. Exosomes are classified as extracellular vesicles released from many cell types, including cancer cells [11]. The intensity of research on microRNAs has significantly expanded our knowledge on this subject, clearly suggesting their enormous biological importance. This leads to the discovery of increasingly new non-coding microRNAs. In 2019, over 1900 microRNAs were classified in the microRNA database, miRBase [12]. Microarray analyses of microRNA expression modulation in microRNA-transfected cells have shown that multiple microRNAs can interact with the same microRNA molecule and that a single microRNA molecule affects multiple targets, directly affecting the amount of translated proteins. This demonstrates that microRNAs play a crucial function in regulating many metabolic processes [13]. The formation of microRNAs begins with the transcription of specific informational subunits by RNA polymerase II to form final functional regulators in subsequent stages of maturation. Most microRNAs are produced by the canonical pathway, but there are also products of the non-canonical pathway. Binding of the 3′ UTR of mRNA leads to modulation or translation of microRNA, which results in deadenylation of microRNA and translational repression. Scientists have found that microRNAs regulate approximately 60% of microRNA transcripts across species. This control of individual gene expression allows microRNAs to play a crucial role in many metabolic processes, including progression, response to various environmental factors, cell maturation, and apoptosis. Physiologically, the processes of transcription, transformation, and binding of microRNA molecules to obtain sequences complementary to specific mRNA chains with normal function involve the reduction/repression of specific genes. This results from blocking protein translation and stabilizing the altered microRNA, which allows us to observe physiological cell growth, proliferation, differentiation, and apoptosis under appropriate conditions. The fact that microRNAs alter the function of microRNAs controlling all these processes suggests that microRNAs may also play an important role in the process of carcinogenesis [14]. Disturbances in the expression or function of microRNAs can lead to various diseases, such as neurodegenerative disorders, viral infections, autoimmune diseases, metabolic diseases and cancer. There is no doubt that genetic amplification/deletion, methylation of microRNA genomic loci, and dysregulation of primary microRNAs via transcription factors and factors involved in the microRNA biogenesis pathway often result in alterations in microRNA expression and function in different cancers [15]. The mechanisms of microRNA dysregulation underlying carcinogenesis include chromosomal abnormalities, alterations in transcriptional control, epigenetic changes, and defects in the microRNA biogenesis process [16]. In his study, Seyhan used previously described whole-genome sequencing datasets from The Cancer Genome Atlas and computational analysis, demonstrating that over-mutated microRNA genes were commonly found in 30 various cancers and were associated with disease progression and women’s survival [17]. However, microRNAs do not necessarily initiate changes in the modulation of microRNA chain expression, despite their dysregulation. MicroRNA modulation can be caused by the action of various endogenous non-coding RNAs, such as long non-coding RNAs and circular RNAs, which also play a role in cancer development in the post-transcriptional phase. It has been found that these various non-coding RNAs attempt to bind to other microRNAs and exert a “sponge effect” to inhibit further microRNA regulatory activity [18]. Cancer cells produce energy as a result of increased glucose consumption and metabolism via anaerobic glycolysis. Even under conditions of normal oxygenation, aerobic glycolysis plays a minor role. This is the so-called Warburg effect. MicroRNAs participate in different aspects of the cancer cell process. Liu and colleagues demonstrated that in pancreatic cancer, microRNA-3662 inhibits this effect by acting on glycolytic genes and enzymes: lactate dehydrogenase A, platelet phosphofructokinase and pyruvate kinase M [19]. MicroRNAs may be overexpressed or underregulated in cancer cells, which is associated with a number of genetic and epigenetic changes, such as aberrant DNA methylation.

**Figure 2:**
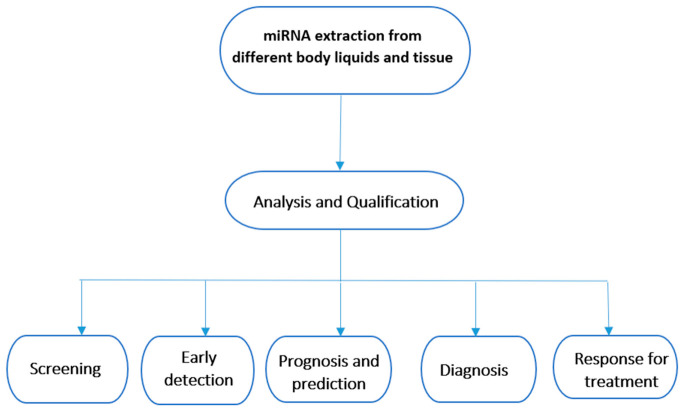
Application of microRNAs as biomarkers for cervical cancer.

Liquid biopsy is a noninvasive method for analyzing tumor products, including those in blood, offering the ability to monitor tumor progression, metastasis, and treatment response in real time. This involves detecting circulating microRNAs. MicroRNAs are stable and resistant to RNase and other factors, such as repeated freeze–thaw cycles or long-term storage. MicroRNAs are easily detectable in various body fluids using highly sensitive or specific methods. Numerous studies indicate that microRNAs can be noninvasive markers of various cancers, including cervical cancer (Figure 2) [20,21,22].

## 3. MicroRNAs as Biomarkers of Cervical Cancer

Unfortunately, most cases of cervical cancer are diagnosed at a late stage, resulting in a relatively high mortality rate among women with this cancer. Therefore, intensive research is necessary to find a trustworthy marker for diagnosing premalignant and early asymptomatic cervical cancers. Liquid biopsy in women with CC is a noninvasive procedure that allows for microRNA profiling, which can provide information on microRNA signatures for various types of cancer at different stages. Thanks to this, we can continuously and over the long term assess the course of the disease and monitor the response to treatment. MicroRNA testing has many advantages, such as the detection of even very small amounts of altered material and high stability in tissues and fluids. Recently, all deregulated microRNAs in tumor cells, plasma, and different fluids in women with cervical cancer have been cataloged, which may enable the development of new diagnostic tools and therapeutic approaches. Defining circulating microRNAs or their specific signatures in biological fluids may be useful in the screening, detection, prognosis, and clinical monitoring of patients undergoing cervical carcinogenesis. Lopez AJG and Lopez JA listed approximately 70 different microRNAs that have been associated with cervical cancer in their meta-analysis. They may participate in the carcinogenesis process at various stages. Changes in microRNA expression were observed at each of the four stages of the carcinogenesis model, but the results showed discrepancies across studies. In the current analysis, we focused on approximately 30 types of microRNAs that are most studied and useful in the diagnosis of CC [23].

The expression of microRNAs in CC is measurable and can decrease or increase. Some microRNAs are significantly upregulated and act as promoters of cancer progression by silencing tumor suppressors, such as apoptosis-related genes. Their expression is essential for tumor suppression and apoptosis; on the other hand, downregulation of such microRNAs may be associated with proliferation, invasion, and metastasis in cancers. Geng et al., using real-time quantitative polymerase chain reaction (RT-qPCR), examined the expression of microRNA-34a in women who tested positive for HPV16 and HPV18 with Cervical Intraepithelial Neoplasia (CIN) 1–3 and CC, and they found a decrease in expression [24]. Wang et al., using the same method, also reported that miR-214 expression was decreased in women with cervical cancer, CIN 1–3 [25]. In turn, Barazandeh et al. found a decrease in expression and showed that there were decreased levels of microRNA Let-7g [26]. However, Farzanehpour et al. demonstrated significantly higher expression of microRNA-192, microRNA-9, and microRNA-205 in tissue samples from tumors than in healthy tissue. Furthermore, the expression of microRNA-192 and microRNA-205 was emphatically higher in the cancer subject compared to the pre-cancer subject. Serum samples showed increased expression levels of these microRNAs in cancer patients compared to healthy individuals. Expression of these three microRNAs (microRNA-9, microRNA-192, and microRNA-205) was significantly higher in the pre-cancer group compared to the healthy group. MicroRNA-205 expression was also significantly higher in the cancer group compared to the pre-cancer group. Receiver operating characteristic (ROC) analyses demonstrated the highest area under the curve (AUC) for microRNA-192. Considering the large increase in microRNA-192 expression in cancer and pre-cancerous tissue and blood compared to healthy tissue and blood, the area under the ROC curve (0.98 for blood) for MicroRNA-192 can be used as a potential biomarker for early detection of cervical cancer [27]. Studies of microRNA expression have shown changes in these expressions at different stages of cervical cancer. According to Yang et al., the expression of microRNA-494 is increased in stage IB CC [28]. However, the levels of microRNA-195 and microRNA-144 are decreased in stage IB [29]. Other studies have shown that the levels of microRNA-375, microRNA-145, and microRNA-124 in stages 1 and 2 were decreased, while the levels of microRNA-99a/b, microRNA-92a, and microRNA-150 were increased. These results suggest that some of the above-mentioned microRNAs can be considered diagnostic and prognostic biomarkers of cancer progression [30,31,32]. In a study by Qiu and colleagues, samples from women with CC and cervical intraepithelial neoplasia were compared with samples from healthy women. They demonstrated that circulating levels of microRNA-21 were significantly higher in women with early-stage CC, while blood microRNA-125b and microRNA-370 were significantly lower. Moreover, the combination of expression of serum microRNA-21, microRNA-125b and microRNA-370 as a microRNA signature shows good performance in identifying early-stage CC from cervical intraepithelial neoplasia and healthy women. Furthermore, high serum levels of microRNA-21 and low serum levels of the other two microRNAs are strongly associated with the occurrence of lymph node metastasis (LNM) and tumor recurrence. Women with high serum microRNA-21, low serum microRNA-125b, and low serum microRNA-370 levels have a higher risk of developing lymph node metastasis and recurrence, whereas patients with low serum microRNA-21 and high serum microRNA-125b and high serum microRNA-370 levels do not experience LNM or recurrence. Combined serum determination of this novel microRNA panel, including these three microRNAs, shows great promise for the detection and monitoring of early-stage cervical cancer [33]. In another study, Chen and colleagues used qRT-PCR to assess the expression of microRNA-34a and microRNA-206 and found they were downregulated in cervical cancer tissue. This resulted in increased expression of the target genes Bcl2 and c-Met, which promotes the development of cervical cancer. Kaplan–Meier and log-rank analyses showed that decreased expression of microRNA-34a and microRNA-206 strongly correlated with shorter overall survival. They also observed that in women with reduced expression of these microRNAs, cancer cells had a greater tendency to metastasize to lymph nodes, and the cancer showed a more advanced histological grade and stage of the disease. According to the researchers, microRNA-34a and microRNA-206 may be potential prognostic and therapeutic tools in the treatment of cervical cancer [34].

Numerous studies have examined the overexpression of microRNA-21 in cervical cancer. However, due to inconsistencies in some results, Gebrie conducted a meta-analysis and systematic review of 53 studies [35]. Numerous studies have consistently demonstrated increased expression of microRNA-21 in cervical carcinogenesis [36,37,38,39,40,41]. MicroRNA-21 has been shown to play a complex role in cancer development and tumorigenesis through a variety of mechanisms. MicroRNA-21 influences the expression of numerous genes, which regulate their downstream signaling pathways, promoting cervical carcinogenesis [42]. Therefore, this type of microRNA possesses oncogenic properties, playing a significant role in the development and progression of cervical malignancy. Furthermore, it is characterized by high diagnostic accuracy in diagnosing cervical cancer. The overall AUC of the summary receiver operating characteristic (SROC) of microRNA-21 as an indicator of diagnostic accuracy for CC was 0.80 (95% CI: 0.75, 0.86). Overexpression of microRNA-21 has also been shown to predict a poorer prognosis in patients with cervical cancer [35,43]. Okoye and colleagues found that the expression of microRNA-21, microRNA-182, let-7b, microRNA-145, and p53 in serum is comparable to their expression in cervical cells, making them useful in differentiating abnormal from healthy cervical tissue. Therefore, the expression of these microRNAs based on Pap smear grade suggests that they could be used to monitor cervical carcinogenesis [44]. Moreover, Liu et al. [37] indicated that the expression of microRNA-21 in cervicovaginal lavage specimens of CC patients was higher than that of the HPV-positive and HPV-negative normal subjects. Differential exosomal microRNA-21 expression was also assessed between the HPV-positive and HPV-negative normal groups. The exosomal miRNA-146a level in cervicovaginal lavage specimens of the CC women was also significantly higher than that of the HPV-positive patients and HPV-negative normal. However, there were no statistically significant changes in the levels of microRNA-21 or microRNA-146a in the supernatant of cervicovaginal lavage in any of the three groups. Additionally, the relative microRNA-21 and microRNA-146a levels in exosomes were much higher than in the supernatant.

New research on the usefulness of microRNA-486-5p in the diagnosis of CC found that the relative expression level of this microRNA in serum in patients with early-stage cancer is almost twice as high as in healthy women. It has a relatively better predictive value for early-stage CC than squamous cell carcinoma antigen (SCC-Ag), characterized by a high AUC (0.865), sensitivity (1.000), and specificity (0.804). These results demonstrate that this test has the potential to become a noninvasive, more effective tool in the early diagnosis of CC compared to previously commonly used biomarkers in the diagnosis of cervical cancer, such as SCC-Ag. Furthermore, overexpression of this microRNA was associated with a high probability of increased maximum tumor diameter, which is a prognostic factor indicating tumor progression and LNM in cancer women. These findings indicate that expression of microRNA-486-5p can also be used in the dynamic monitoring of cervical cancer progression. However, the mechanism of microRNA-486-5p′s involvement in occurrence or progression of CC remains unclear [45].

MicroRNAs are promising biomarkers for cervical cancer in both tissue biopsy- and liquid biopsy-based diagnostics. Profiling microRNAs in cervical tissue can improve diagnostic yield. Tissue biopsy is useful in differentiating normal cervical tissue from dysplasia and cancer. MicroRNA expression in tissue correlates with the progression of CIN stage and tumor type differentiation (squamous cell carcinoma, adenocarcinoma). Specific microRNAs correlate with tumor volume, LNM, risk of recurrence, and overall survival. The most frequently used techniques for determining microRNAs are quantitative reverse transcription PCR (qRT-PCR), microarray analysis, in situ hybridization, and next-generation sequencing (NGS).

Liquid biopsy, in turn, involves the examination of circulating microRNAs, allowing for early detection of CC, monitoring cancer progression, and predicting resistance to therapy. It can also complement screening tests by improving sensitivity and specificity [46]. A comparison of tissue and liquid biopsy is shown in Table 1.

A new trend is also the determination of microRNAs in exosomes. This test allows us to better reflect the biology of the tumor and increase sensitivity.

The etiological factor of cervical cancer is HPV infection. HPV oncoproteins (E5, E6, and E7) can stimulate carcinogenesis through various cellular pathways [47]. Furthermore, these proteins directly or indirectly alter the expression of many microRNAs, modulating individual phases of the viral life cycle. Several studies have attempted to correlate microRNA expression levels with human papillomavirus genotypes. For example, Li et al. demonstrated that microRNA-218 expression levels are elevated in women with cervical intraepithelial neoplasia infected with high-risk HPV compared to those infected with low- or intermediate-risk HPV. High-risk HPV infections have been found to result in lower microRNA-218 expression, contributing to the pathogenesis of cervical cancer [48]. McBee et al. also observed decreased expression of microRNA-218 and microRNA-433 and increased expression of microRNA-16, microRNA-21, microRNA-106b, and microRNA-135b in patients with HPV-16-positive CC [49]. Numerous studies conducted on HPV-positive human cervical cancer cell lines (such as the CaSki and SiHa cell lines) have shown that microRNA-182 is overexpressed [50,51,52]. Higher expression of this microRNA has also been demonstrated in the serum of HPV-positive patients. The overexpression of microRNA-182 in the serum of women with positive cervicitis and pre-cancerous lesions suggests that it is a potentially less invasive biomarker for early disease detection. A significant correlation was also found between microRNA-182 and microRNA-200c at the cellular level. In invasive and metastatic cervical cancer, increased expression of microRNA-200c was observed in the cervix of affected women. A three-fold increase in the concentration of micro-200c in HPV-positive people compared to HPV-negative people supports its usefulness in testing high-risk groups [53]. Farzanehpour et al. also confirmed the activation of microRNA-9 and microRNA-192 by HPV in serum and tumor tissue, making these microRNAs potential biomarkers for squamous cell papillomavirus-induced cancers [27]. Distinguishing the role of microRNAs in carcinogenesis and the associated inflammatory process in HPV infection remains an open question and requires further research. Selected microRNAs useful in CC diagnostics are presented in Table 2.

## 4. Clinical Utility of microRNA in Cervical Cancer

Currently, standard CC screening methods include cytology, HPV DNA testing, and serum squamous cell carcinoma antigen. MicroRNA-based tests can complement or replace these conventional methods. Current research suggests that some single microRNAs or panels of microRNAs can achieve diagnostic performance (sensitivity, specificity) comparable to or better than cytology, although HPV testing generally still provides the highest sensitivity for detecting high-grade cervical intraepithelial neoplasia (CIN2/CIN3). Conventional cytology has moderate sensitivity but relatively good specificity. The advantages of cytology are its low cost and method validation. However, its disadvantages include low sensitivity for adenocarcinoma and the need for repeated testing. DNA HPV testing is the most sensitive screening method but has a lower positive predictive value (PPV) than cytology because HPV infections are often transient. SCC-Ag is of little use in screening and is primarily used for monitoring and prognostic purposes. It demonstrates low sensitivity in early-stage cancer.

Studies using microRNAs have demonstrated strong discrimination between cancer patients and healthy women. However, panels generally outperform single microRNAs. Tamenang et al. found that a combination of three microRNAs (microRNA-21, microRNA-205, and microRNA-218) significantly differed across patient cohorts. The combination of these three markers demonstrated the best diagnostic value for high-grade squamous cell intraepithelial lesions (sensitivity 79% and specificity 71%) and cancer (sensitivity 90% and specificity 65%). This combination demonstrates high diagnostic performance for multiple HPV infections (HPV16/18) [43].

In summary, current research suggests that selected microRNA panels may demonstrate sensitivity/specificity comparable to or superior to cytology and SCC-Ag, but they have not yet surpassed HPV testing in robust, extensive clinical validation. The combination of microRNAs, cytology, and HPV DNA testing may also be important. The sensitivity and specificity values of cervical cancer markers are presented in Table 3.

## 5. Conclusions and Future Perspectives

Cervical cancer, with a high morbidity and mortality rate, poses a major challenge to medicine and requires effective tools in the form of markers for the early detection of this disease. The general view is that cervical cancer research requires effective, large-scale, randomized prospective trials. Although numerous commonly used oncological biomarkers, such as carcinoembryonic antigen (CEA) and SCC-Ag, have been identified in patient blood, their specificity and sensitivity for cervical cancer are limited due to their specificity and sensitivity for cervical cancer [54,55]. Describing the aberrant expression of various types of microRNAs in CC has identified new molecular mechanisms of tumorigenesis in this tumor and created significant opportunities for translating microRNA research into the clinical setting [56]. MicroRNAs are important regulators of many mechanisms associated with cervical cancer, such as proliferation, invasiveness, apoptosis deregulation, and metastasis [57]. Numerous studies highlight the potential usefulness of microRNAs in the diagnosis and treatment of cervical cancer [58,59,60]. The diagnostic role of microRNAs is perhaps one of the most attractive and modern methods used in oncology. Recent studies have made significant progress in microRNA profiling in liquid biopsy samples and clearly emphasize the importance of specific microRNA signatures in the diagnosis/prognosis of cervical cancer [61,62]. Some types of microRNAs possess oncogenic potential, exhibit differential expression in various stages of CC, and may be associated with high-risk HPV infection, making them promising biomarkers in cancer screening and monitoring for cancer recurrence [3]. Careful analysis of specific microRNA panels indicates high diagnostic value, including high sensitivity and specificity in patients with cervical cancer lesions. Interestingly, circulating microRNAs are valuable biomarkers for early-stage cervical cancer and for monitoring the clinical outcome of advanced cancer, given that the majority of cancer-related deaths are caused by metastases to distant organs [63]. Many microRNAs have been described as potential biomarkers that can distinguish women with early-stage CC from healthy subjects without cancer or patients with mild disease by testing a small amount of blood [6,33,64]. Focusing on microRNAs involved in the deregulation of biological pathways leading to cervical cancer carcinogenesis may be a logical approach to developing a minimally invasive biomarker test for early detection of CC and may reduce the need for invasive cervical biopsies. It should be emphasized, however, that extensive research is needed to validate microRNA tests as a diagnostic biomarker compared to cytological methods and screening tests for squamous cell papillomavirus infection. Several molecules capable of targeting oncogenic microRNAs have been developed, such as synthetic antisense oligonucleotides encoding sequences complementary to microRNAs [6].

Due to the association of microRNAs with therapy resistance in cervical tumors, there is a potential for the development of new therapies using microRNA inhibitors or as a supplement to chemotherapy and radiotherapy. Hypomethylating agents, such as decitabine or 5-azacytidine, induce epigenetic silencing of microRNAs and reduce the expression of several microRNAs in tumors, which may represent an effective new therapeutic approach [65].

A limitation of microRNA use is unsatisfactory validation due to the lack of standardized normalization procedures, varying methodologies, and the difficulties of differentiation between similar microRNAs. Specialized and dedicated analytical tools are also necessary to detect small microRNAs [66]. In addition, microRNAs can be released from erythrocytes or white blood cells during storage, which can lead to erroneous results. Moreover, white blood cells and erythrocyte hemolysis may also alter the quality and quantity of extracted microRNA. The level of microRNAs in the blood depends on many factors, including age, sex, habits and lifestyle (diet, stimulants). Knowledge about the influence of a given factor on microRNA allows for better interpretation of the diagnostic values of microRNAs in a specific environment [67]. A significant factor determining the use of microRNAs as a diagnostic tool is the correlation of microRNAs in people with different types of cancer. For example, elevated blood microRNA concentrations have been detected not only in patients with cervical cancer but also in patients with cancer of the pancreas, esophagus, liver, and breast, as well as colorectal cancer [68,69,70,71]. A separate problem is the lack of consistency in results, even in similar studies of the same cancer. Reduced microRNA expression in tumors can be caused by genomic and epigenomic abnormalities, but in the circulation, this only occurs when the tumor adversely affects microRNA expression in other cells or reduces the stability of circulating microRNAs. This suggests that reduced serum microRNA levels can be considered a nonspecific response to the presence of cancer [72]. It is advantageous that microRNA determination using liquid biopsy can encompass other body fluids that may contain cancer-specific microRNAs [73].

The significant role of microRNAs in cervical cancer diagnosis in the coming years may involve predicting the progression of CIN to invasive cancer and triaging HPV-positive patients.

Despite intensive research on the use of microRNA in the detection of CC, it is still not a standard test due to its limitations. These include lack of standardization at many stages: extraction methods, normalization control, cut-off thresholds, and methodological differences. Moreover, most studies are single-center and often retrospective, and there are no multicenter studies with adequate prospective validation. Ethnic variation also exists. Costs should also be considered, as tests based on qRT-PCR and sequencing are expensive.

Research on the diagnostic utility of microRNAs in the early diagnosis and prognosis of various solid tumors (including cervical cancer) should continue, and new approaches might be developed that can improve treatment efficacy and patient survival. Therefore, intensified efforts are needed to apply microRNAs clinically as biomarkers. The primary focus should be on addressing existing methodological and analytical challenges, utilizing standard operating procedures, automated and standardized assays, and miniaturization of methods to improve inter- and intra-method reproducibility. Independent interdisciplinary collaboration is crucial, encompassing all fields of medicine—both diagnostics (radiology, laboratory medicine, and rheumatology) and therapeutic areas (surgery, gynecology, and oncology)—and is essential throughout the entire process of cervical cancer diagnosis and treatment.

Future diagnostic strategies for cervical cancer, including microRNA testing, may be expanded to include circulating tumor DNA (ctDNA) and DNA methylation markers. However, further large-scale prospective studies and standardized protocols are needed to make microRNA a routine method in clinical practice for various cancers, including cervical cancer.

## Figures and Tables

**Table 1:** Tissue biopsy vs. liquid biopsy.

Feature	Tissue Biopsy	Liquid Biopsy
Tumor specificity	High	Moderate
Invasiveness	Invasive	Minimally invasive
Early detection potential	Moderate	High
Dynamic monitoring	Limited	Excellent
Tumor heterogeneity assessment	Limited	Better systemic representation
Sampling frequency	Low	High

**Table 2:** Selected microRNAs in cervical cancer.

Symbol	Expression	Materials	Biomarker
microRNA-34a	Down	Tissue	Prognosis, monitoring, response to treatment
microRNA-9	Up	Tissue, serum	Diagnosis, prognosis
microRNA-20b	Up	Tissue	Prognosis, monitoring, prediction of survival
microRNA-16	Up	Tissue	Diagnosis, HPV infection
microRNA-92a	Up	Plasma	Diagnosis, prognosis
microRNA-200c	Up	Whole blood	Early diagnosis, prognosis
microRNA-99a	Up	Whole blood	Diagnosis, prognosis
microRNA-let-7g	Down	Serum	Early detection, prognosis, monitoring
microRNA-124	Down	Plasma	Diagnosis, prognosis
microRNA-let-7b	Up	Serum	Early diagnosis
microRNA-125b	Down	Serum	Early detection prognosis, recurrences, metastasis, prediction of survival
microRNA-135b	Up	Serum	Diagnosis, prognosis, HPV infection
microRNA-144	Down	Whole blood	Early detection, prognosis
microRNA-145	Down	Serum	Early detection, prognosis, monitoring, prediction of survival
microRNA-146a	Up	Tissue	Diagnosis, HPV infection
microRNA-150	Up	Plasma	Diagnosis, prognosis
microRNA-182	Up	Serum	Early detection, prognosis, monitoring
microRNA-192	Up	Tissue, serum	Diagnosis, prognosis
microRNA-195	Down	Whole blood	Early detection, prognosis
microRNA-205	Up	Serum	Diagnosis, prognosis, prediction of survival
microRNA-203a	Up	Serum	Prognosis, monitoring, prediction of survival
microRNA-206	Down	Tissue	Diagnosis, prognosis, response to treatment
microRNA-208	Up	Plasma	Early detection, HPV infection
microRNA-214	Down	Serum	Diagnosis, prognosis
microRNA-218	Down	Serum	Early detection, prognosis, HPV infection
microRNA-21	Up	Serum	Early detection, prognosis, prediction of survival
microRNA-370	Down	Serum	Early detection, prognosis, metastasis, recurrence
microRNA-375	Down	Serum	Early detection, prognosis
microRNA-425-5p	Up	Serum	Prognosis, monitoring, prediction of survival
microRNA-486-5p	Up	Serum	Diagnosis, prognosis, monitoring
microRNA-494	Up	Serum	Early detection
microRNA-433	Down	Serum	Early detection, monitoring, HPV infection
microRNA-106b	Up	Serum	Monitoring recurrences, HPV infection
microRNA-1290	Up	Serum	Early detection, prognosis, prediction of survival

**Table 3:** Comparison of the diagnostic sensitivity and specificity of cervical cancer detection methods.

Test	Sensitivity (%)	Specificity (%)	Main Role
Conventional Pap smear	50–75	85–95	Standard screening
Liquid-based cytology	76–84	49	Standard screening
HPV DNA testing	90–95	85–95	Primary screening
SCC-Ag	30–70	70–90	Monitoring/prognosis
microRNA-21	70	78	Experimental biomarker
microRNA-205	90	59	Experimental biomarker
microRNA208	70	74	Experimental biomarker
microRNA-21, microRNA-205, microRNA-208	90	65	Potential future adjunct

## Data Availability

No new data were created or analyzed in this study. Data sharing is not applicable to this article.
